# Age-specific Risk of Herpes Zoster in Immunocompetent Adults ≥18 Years-of-age—A Retrospective Cohort Study in the United States

**DOI:** 10.1093/ofid/ofag153

**Published:** 2026-03-28

**Authors:** Rachel A Cohen, Driss Oraichi, Agnes Mwakingwe-Omari, Bruno Anspach, Desmond Curran, Mitra Yousefi, Huifeng Yun

**Affiliations:** GSK, Rockville, Maryland, USA; GSK, Rockville, Maryland, USA; GSK, Rockville, Maryland, USA; GSK, Rockville, Maryland, USA; GSK, Wavre, Belgium; GSK, Rockville, Maryland, USA; GSK, Rockville, Maryland, USA

**Keywords:** adult, herpes zoster, immunocompetent, incidence, United States

## Abstract

**Background:**

Approximately one-third of United States (US) individuals develop herpes zoster (HZ). Recent HZ incidence data are lacking, especially in immunocompetent adults 18–49 years-of-age (YoA). Recombinant HZ vaccine is recommended for US adults ≥50 YoA and immunocompromised/immunosuppressed adults ≥19 YoA.

**Methods:**

This retrospective cohort study (2015–2022, Merative MarketScan Commercial/Medicare) estimated age-specific HZ burden and incidence rates (IRs) per 1000 person-years in immunocompetent adults (≥18 YoA), and calculated HZ adjusted IR ratios (aIRRs) versus 50–59 YoA. Using the predetermined noninferiority margin of 0.62, HZ aIRRs versus 50–59 YoA were significantly higher (95% confidence interval lower limit [95% CI LL] > 1.0), comparable (95% CI LL >0.62 and ≤1.0), or inconclusive (other result). HZ definition: International Classification of Diseases (ICD-10) code B02.2x plus antiviral within 7 days (only ICD-10 in sensitivity analysis). Immunocompetent definition: no comorbidities (ie, asthma, chronic kidney disease, chronic obstructive pulmonary disease, diabetes, depression, stress, and trauma) or immunocompromising/autoimmune disease or medication.

**Results:**

In this immunocompetent population without comorbid conditions (57% of adults overall), HZ IR increased with age, from 0.77 (18–29 YoA) to 4.67 (≥80 YoA). HZ risk was comparable in 30–39 YoA (aIRR 0.66 [95% CI 0.64–0.68]) and 40–49 YoA (0.75 [0.73–0.77]) versus 50–59 YoA. Proportions with postherpetic neuralgia (from 40 to 49 YoA) and HZ hospitalization (from 60 to 69 YoA) tended to increase with age. In sensitivity analyses using a broader HZ definition, HZ burden was ∼30% higher.

**Conclusions:**

The study identified a substantial HZ burden in younger immunocompetent adults (30–49 YoA) without comorbid conditions, with statistically comparable IRs versus 50–59 YoA.

## Teaser

This United States retrospective study identified a previously under-recognized substantial herpes zoster incidence in younger immunocompetent adults (30–49 years-of-age) without comorbid conditions. The findings may contribute to discussions on the potential need for earlier vaccination.

## Graphical Abstract

**Graphical Abstract:**
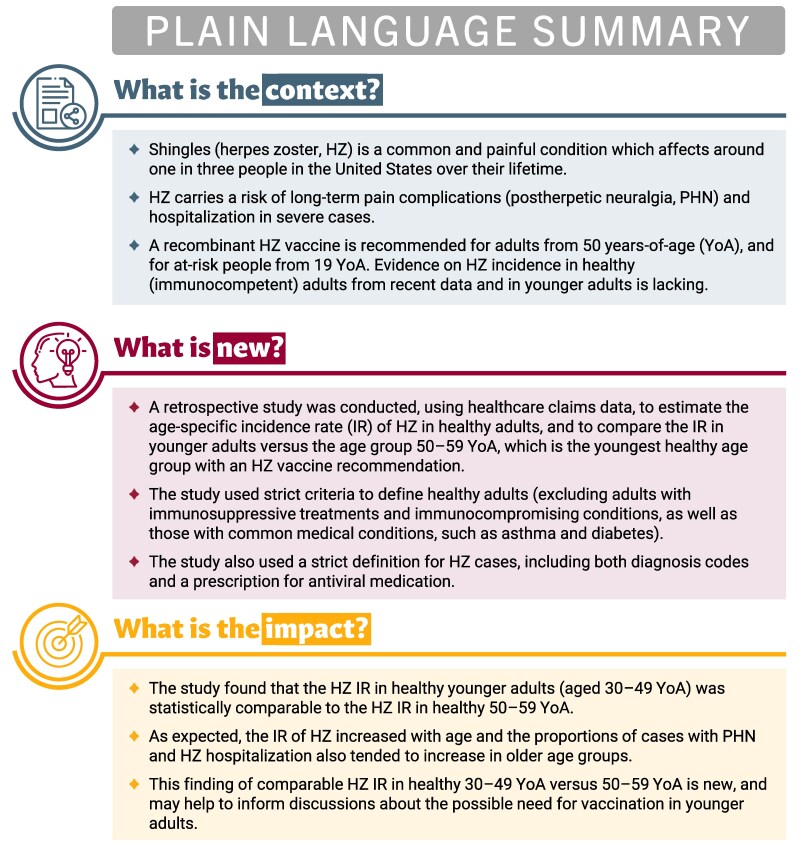

Herpes zoster (HZ, shingles) is caused by reactivation of the latent varicella-zoster virus (VZV), which initially causes varicella (chickenpox) [1]. HZ is characterized by a painful maculopapular rash that progresses to vesicular lesions. Prodromal symptoms, including pain, itching, and tingling, may precede the rash, which typically resolves within 2–4 weeks. The most common complication of HZ is postherpetic neuralgia (PHN), defined as persistent pain for ≥90 days after rash onset, which can last for years [1]. Overall, 10%–18% of individuals with HZ develop PHN [1], and the risk increases with age, from ∼20% in individuals 60–65 years-of-age (YoA) to over 30% in individuals ≥80 YoA [2]. Other HZ complications include vision or hearing loss, encephalitis, and pneumonia [1]. Among immunocompetent adults (≥50 YoA) with HZ, hospitalization occurs in 0.9% overall, increasing with age to 2.8% in adults ≥80 YoA [3]. There is an evidence gap on HZ burden in younger immunocompetent age groups.

In the United States (US), approximately one-third of individuals will develop HZ during their lifetime, with an estimated annual incidence of 1 million cases [1]. HZ incidence increases with age, as VZV-specific cell-mediated immunity declines from around 50 YoA [4, 5]. Studies have also shown temporal trends in HZ incidence with increases over time [6] for example, incidence rate (IR) per 1000 person-years (PY) increased in US adults ≥65 YoA from 10.0 to 13.9 between 1992 and 2010 [7].

A live-attenuated HZ vaccine was recommended in the US for adults ≥60 YoA in 2006 [8]. Since 2017, recombinant HZ vaccine (RZV) is recommended for adults ≥50 YoA and, since 2021, for adults ≥19 YoA at increased risk of HZ because of immunodeficiency or immunosuppression [9]. Prior to 2017, the US HZ IR per 1000PY was 3.4 (18–49 YoA) to 8.4 (≥65 YoA) among all individuals [10]. In the immunocompetent US population, HZ incidence data are available from 2011 to 2015, showing IRs (per 1000PY) of 7.2 in 50–54 YoA increasing to 14.0 in ≥80 YoA [3]. More recent HZ incidence data in the US immunocompetent population [3] and previous studies generally focused on adults ≥50 YoA, given the recommended vaccination age. Prior studies that explored HZ incidence in immunocompetent populations also did not have robust exclusion criteria for comorbid conditions, leading to inclusion of populations with comorbid conditions at higher risk of HZ [11], and possibly leading to misclassification of the immunocompetent population.

The objective of this retrospective cohort study (from 2015 to 2022) was to estimate HZ IRs in immunocompetent adults, defined using stringent exclusion criteria for comorbid conditions, in approximate 10-year age groups. Additionally, the risk of HZ by age groups within 18–49 YoA was compared with the risk in the 50–59 YoA immunocompetent population without comorbid conditions ie, a subgroup of the ≥50 YoA immunocompetent population, representing the youngest immunocompetent age group for whom vaccination with RZV is currently recommended. The proportions of adults with HZ who developed PHN or that were hospitalized were also evaluated. A plain language summary of the study is provided.

## METHODS

### Study Design and Population

A retrospective cohort study was conducted using the Merative MarketScan Commercial and Medicare Supplemental insurance databases (from 1 October 2015 to 31 March 2022), to estimate age-specific IRs per 1000PY of HZ in immunocompetent adults. The Merative MarketScan Commercial Database is a nationally representative data sample of the US population with employer-sponsored health insurance. The database contains deidentified individual patient-level data and medical service (inpatient, outpatient, pharmacy, enrollment, and laboratory tests) claims for all settings of care among individuals from 120 contributing employers and 40 unique contributing health plans [12].

The overall study population included adults ≥18 YoA, with no history of HZ or HZ vaccination, and with 15 months continuous enrollment (considering a grace period of 45 days) in the database (baseline period). Following the baseline period, the index date was the first day of the 16th month of continuous enrollment. Participants were followed until the first occurrence of a censoring event: occurrence of HZ, HZ vaccination, loss of coverage, or end of study (31 March 2022).

From the overall study population, participants were excluded in 3 steps to arrive at the immunocompetent population. (1) Participants were excluded with specific immunocompromising conditions (IC) ie, human immunodeficiency virus, hematologic malignancy, stem cell transplant, solid organ transplant, and solid tumors, or specific autoimmune diseases (AIDs) ie, inflammatory bowel disease, multiple sclerosis, psoriatic arthritis, psoriasis, rheumatoid arthritis, and systemic lupus erythematosus. (2) Participants with the following comorbid conditions were excluded: asthma, chronic kidney disease, chronic obstructive pulmonary disease, diabetes mellitus, depression, stress, and trauma. (3) Among the population without comorbid conditions, participants with other IC/AIDs or on immunosuppressive medication were excluded, leaving the immunocompetent population. Validated algorithms were used to identify the IC [13], AID [14–18], and comorbidity populations [19–24]. In addition to the censoring events defined for the overall population, follow-up for individuals in the immunocompetent population was censored if they no longer met immunocompetent criteria (ie, at first occurrence of any specified IC/AID condition, comorbid condition, or other IC/AID or immunosuppressive medication).

HZ events were defined using the International Classification of Diseases 10th revision (ICD-10) codes for HZ (B02.2x) from hospital, emergency department, or ambulatory visit diagnoses, plus dispensing of oral antivirals (acyclovir, valacyclovir, or famciclovir) within 7 days before or after HZ diagnosis. PHN events were defined using ≥1 subsequent diagnosis code B02.2x (any position) in the 90–180 days after HZ, and ≥1 of the following: (1) ≥ 1 incident dispensing for anti-PHN medications in the 0–60 days after the first HZ diagnosis without an anti-PHN medication in this medication class in the 365 days prior to initial HZ; (2) an ICD-10 diagnosis B02.2x (HZ with other nervous system involvement) in the 90–180 days after an HZ event; or (3) a new ICD-10 diagnosis M79.2 (neuralgia and neuritis, unspecified) or M54.10 (radiculopathy, site unspecified) in the 0–180 days after HZ, without neuralgia or radiculopathy in the 365 days prior to HZ. HZ hospitalizations were defined as inpatient hospitalization with an HZ diagnosis.

Age was treated as a time-varying variable and recalculated annually, with participants assigned to subsequent age groups as necessary. Analyses were stratified by the following approximate 10-year age groups: 18–29, 30–39, 40–49, 50–59, 60–69, 70–79, and ≥80 YoA.

### Analysis

The baseline characteristics of the immunocompetent population were described overall and by age group. Categorical variables were presented as absolute numbers and percentages. Continuous variables were presented as the mean with standard deviation (SD) and/or median with interquartile ranges.

The age-specific IR was calculated using the number of incident cases of HZ divided by the total PY at risk. Person-time at risk was defined as the total follow-up time starting from the index date until the occurrence of the outcome of interest or another censoring event. Exact Poisson 95% confidence intervals (CIs) of the age-group-specific IRs were computed.

The HZ IRs in each age group <50 YoA were compared with the HZ IR in the immunocompetent population 50–59 YoA without comorbid conditions (reference group). Incidence rate ratios (IRRs) were calculated for the comparison of HZ IRs in age groups <50 YoA with the reference group. Adjusted IRRs (aIRRs) and 95% CIs were estimated using Poisson models, and covariates for adjustment included age, sex, number of hospitalizations, region, health plan type, medication, and comorbidities.

Using the predetermined noninferiority margin of 0.62, as defined in a previous study [25], the IR of HZ for each cohort was classified as significantly higher (lower limit of the IRR 95% CI >1.0), comparable (ie, noninferior, lower limit of the IRR 95% CI >0.62 and ≤1.0), or inconclusive (any other result) compared with the reference group (50–59 YoA immunocompetent population). The noninferiority margin of 0.62 in the prior study was based on the ratio of the HZ IR in 50–59 YoA (6.7) and in 60–69 YoA (10.8). At the time, HZ vaccination was recommended by the US Centers for Disease Control and Prevention (CDC) from 60 YoA, and the US Food and Drug Administration (FDA) had approved live-attenuated HZ vaccination for adults 50–59 YoA [25].

The proportion of HZ cases who developed PHN was determined by dividing the number with a first PHN event by all HZ cases with ≥180 days of continuous enrollment following HZ. The proportion of hospitalized HZ cases was determined by dividing the number with a first HZ hospitalization (HZ date from hospital visit only) by all HZ cases (HZ date from hospital, emergency department, or ambulatory visit) diagnosed from the index date until the censoring event. The proportions of HZ cases who developed PHN or had HZ hospitalizations were stratified by age group.

Analyses were conducted in SAS version 9.04.01 (SAS Institute Inc.).

### Sensitivity Analyses

In sensitivity analyses, a less stringent definition of HZ was applied, using only ICD-10 codes to define HZ cases, instead of ICD-10 codes plus antiviral medication prescriptions. The proportions of HZ cases with PHN and HZ hospitalization were also assessed in this HZ population.

A COVID-19 sensitivity analysis was conducted including data up to 1 March 2020, to assess the robustness of the findings after excluding cases during the COVID-19 pandemic. This was undertaken as healthcare systems and hospital visits were impacted by the COVID-19 pandemic, and as an increased HZ risk was observed with COVID-19 infections [26].

## RESULTS

### Population and Baseline Characteristics

The overall population included 20 673 677 adults ≥18 YoA, with no history of HZ illness or HZ vaccination, and with 15 months continuous enrollment in the database. The immunocompetent population comprised 11 827 771 adults (57.2% of overall population), after excluding adults with prespecified IC/AIDs in step 1 (0.5%/1.7% of overall), with comorbid conditions in step 2 (15.3% of overall), and with other IC/AIDs or on immunosuppressive medication in step 3 (25.4% of overall) (see Supplementary Figure 1).

In the immunocompetent population at baseline, mean age was 39.6 years (SD 14.5) and 53.7% were male. During the 15-month baseline period, few had been hospitalized (1.8%) or had COVID-19 (0.6%), and 37.7% had used preventive cancer screening services. The most common comorbidities were hypertension (11.8%) and hyperthyroidism (3.3%) (Table 1; comorbidity data not shown).

**Table 1.** Baseline Characteristics—Immunocompetent Population

	Age Group (years)							
	18–29	30–39	40–49	50–59	60–69	70–79	≥80	Overall
*N* (%), at index date	3 629 744 (30.7)	2 659 597 (22.5)	2 346 668 (19.8)	2 029 277 (17.2)	908 397 (7.7)	160 150 (1.4)	93 938 (0.8)	11 827 771 (100)
Mean age (SD), years	23.6 (3.3)	34.4 (2.9)	44.5 (2.9)	54.4 (2.8)	62.8 (2.3)	73.8 (2.8)	86.3 (5.1)	39.6 (14.5)
Sex, *n* (%)	…	…	…	…	…	…	…	…
Female	1 605 885 (44.2)	1 197 609 (45.0)	1 091 029 (46.5)	969 658 (47.8)	456 796 (50.3)	88 969 (55.6)	62 400 (66.4)	5 472 346 (46.3)
Male	2 023 859 (55.8)	1 461 988 (55.0)	1 255 639 (53.5)	1 059 619 (52.2)	451 601 (49.7)	71 181 (44.4)	31 538 (33.6)	6 355 425 (53.7)
*N* hospitalizations, *n* (%)								
0	3 570 998 (98.4)	2 585 618 (97.2)	2 319 829 (98.9)	2 003 712 (98.7)	891 833 (98.2)	154 673 (96.6)	87 995 (93.7)	11 614 658 (98.2)
1	54 657 (1.5)	70 964 (2.7)	25 021 (1.1)	23 376 (1.2)	15 035 (1.7)	5055 (3.2)	5398 (5.7)	199 506 (1.7)
2	3163 (0.1)	2664 (0.1)	1541 (0.1)	1909 (0.1)	1372 (0.2)	380 (0.2)	489 (0.5)	11 518 (0.1)
≥3	926 (0.0)	351 (0.0)	277 (0.0)	280 (0.0)	157 (0.0)	42 (0.0)	56 (0.1)	2089 (0.0)
Health plan type, *n* (%)								
Commercial	3 629 722 (100)	2 659 548 (100)	2 346 460 (100)	2 027 910 (99.9)	800 874 (88.2)	0	0	11 464 514 (96.9)
Medicare	22 (0.0)	49 (0.0)	208 (0.0)	1367 (0.1)	107 523 (11.8)	160 150 (100)	93 938 (100)	363 257 (3.1)
Geographical region, *n* (%)								
North central	719 175 (19.8)	489 440 (18.4)	432 886 (18.4)	397 167 (19.6)	210 796 (23.2)	66 708 (41.7)	37 784 (40.2)	2 353 956 (19.9)
South	1 668 734 (46.0)	1 211 002 (45.5)	1 088 866 (46.4)	914 470 (45.1)	386 340 (42.5)	43 770 (27.3)	25 368 (27.0)	5 338 550 (45.1)
West	647 964 (17.9)	513 400 (19.3)	437 988 (18.7)	359 499 (17.7)	150 657 (16.6)	18 867 (11.8)	12 478 (13.3)	2 140 853 (18.1)
Northeast	577 984 (15.9)	435 665 (16.4)	376 739 (16.1)	349 928 (17.2)	157 357 (17.3)	30 338 (18.9)	17 833 (19.0)	1 945 844 (16.5)
Missing/unknown	15 887 (0.4)	10 090 (0.4)	10 189 (0.4)	8213 (0.4)	3247 (0.4)	467 (0.3)	475 (0.5)	48 568 (0.4)
Preventive services use, *n* (%)	1 147 115 (31.6)	980 221 (36.9)	953 108 (40.6)	914 461 (45.1)	397 353 (43.7)	46 225 (28.9)	15 331 (16.3)	4 453 814 (37.7)
COVID-19 diagnosis, n (%)	25 130 (0.7)	18 191 (0.7)	12 124 (0.5)	8158 (0.4)	2520 (0.3)	371 (0.2)	168 (0.2)	66 662 (0.6)
Index date by year, *n* (%)								
2017	1 916 962 (52.8)	1 328 256 (49.9)	1 345 954 (57.4)	1 259 648 (62.1)	599 738 (66.0)	115 025 (71.8)	74 007 (78.8)	6 639 590 (56.1)
2018	512 490 (14.1)	394 642 (14.8)	305 075 (13.0)	237 343 (11.7)	90 503 (10.0)	6763 (4.2)	2679 (2.9)	1 549 495 (13.1)
2019	504 620 (13.9)	397 162 (14.9)	312 801 (13.3)	250 714 (12.4)	108 152 (11.9)	21 889 (13.7)	10 242 (10.9)	1 605 580 (13.6)
2020	336 614 (9.3)	260 300 (9.8)	184 983 (7.9)	136 822 (6.7)	51 654 (5.7)	2058 (1.3)	606 (0.6)	973 037 (8.2)
2021	323 762 (8.9)	249 078 (9.4)	179 419 (7.6)	132 590 (6.5)	53 458 (5.9)	14 232 (8.9)	6348 (6.8)	958 887 (8.1)
2022	35 296 (1.0)	30 159 (1.1)	18 436 (0.8)	12 160 (0.6)	4892 (0.5)	183 (0.1)	56 (0.1)	101 182 (0.9)

Abbreviations: COVID-19, coronavirus disease 2019; *n*/*N*, number; SD, standard deviation.

### HZ Incidence Rates in Adults

**Figure 1.**
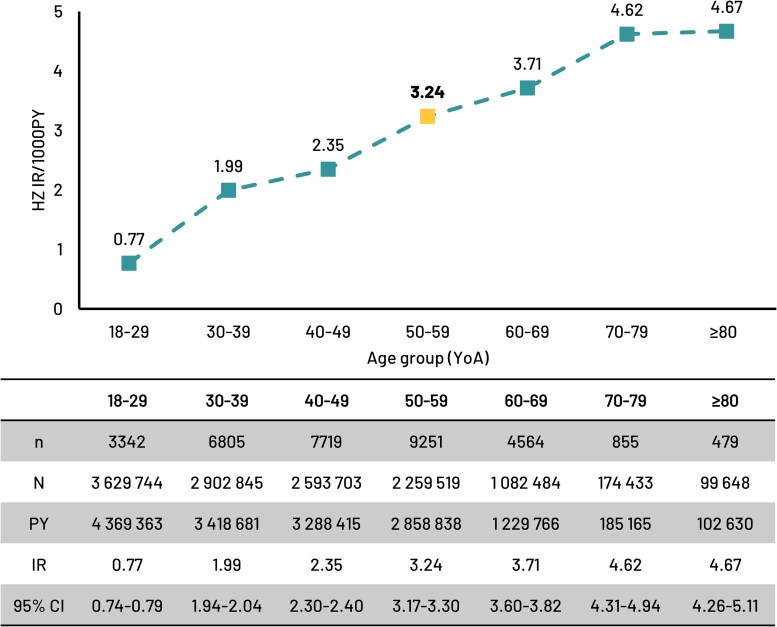
HZ incidence rate per 1000PY (95% CI) in the immunocompetent population by age group. Abbreviations: 95% CI, 95% confidence interval; HZ, herpes zoster; IR, incidence rate; *n*/*N*, number with HZ/total number in age group; PY, person-years; YoA, years-of-age.

Overall, 33 015 HZ events were reported in the immunocompetent population (all ages). The HZ IR increased with age: from 0.77 (95% CI 0.74; 0.79) in 18–29 YoA; 1.99 (1.94; 2.04) in 30–39 YoA; 2.35 (2.30; 2.40) in 40–49 YoA; to 3.24 (3.17; 3.30) in the 50–59 YoA reference group (Figure 1).

### HZ Incidence Rate Ratios

**Figure 2.**
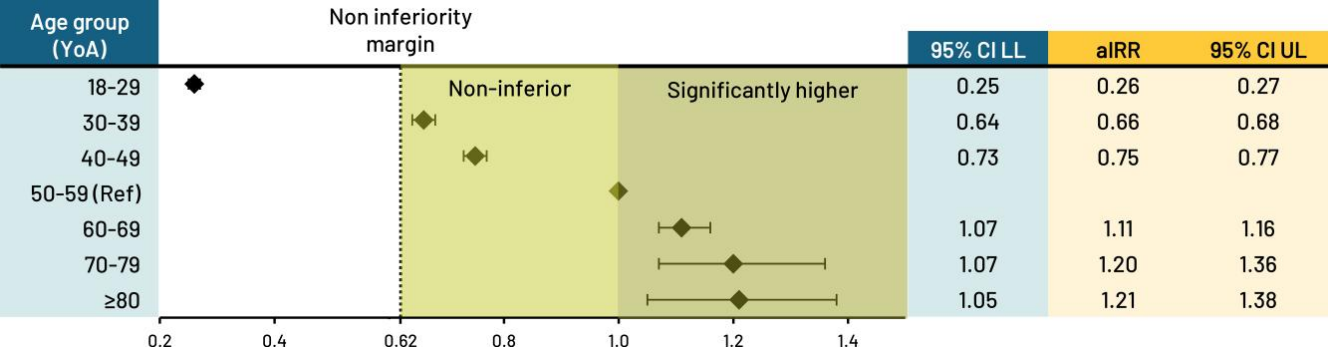
HZ aIRRs (95% CIs) in the immunocompetent population by age group compared with the reference group (50–59 YoA). Abbreviations: 95% CI, 95% confidence interval; aIRR, adjusted incidence rate ratio; HZ, herpes zoster; LL, lower limit; Ref, reference; UL, upper limit; YoA: years-of-age.

Compared with the reference group (50–59 YoA), and given the predetermined noninferiority margin of 0.62 for the aIRR 95% CI lower limit (95% CI LL), the risk of HZ was found to be comparable (ie, the aIRR 95% CI LL >0.62 and ≤1.0) in the immunocompetent population 30–39 YoA with 0.64 95% CI LL (aIRR 0.66 [95% CI 0.64–0.68]) and 40–49 YoA with 0.73 95% CI LL (aIRR 0.75 [95% CI 0.73–0.77]) (Figure 2). Regarding absolute numbers of HZ cases, there were 6805 and 7719 HZ cases in the 30–39 and 40–49 YoA groups, respectively, compared with 9251 in the reference group (Figure 1).

### PHN and HZ Hospitalization in Adults With HZ

Of the 33 015 HZ cases in the immunocompetent population, 396 developed PHN and 70 were hospitalized with HZ.

**Figure 3.**
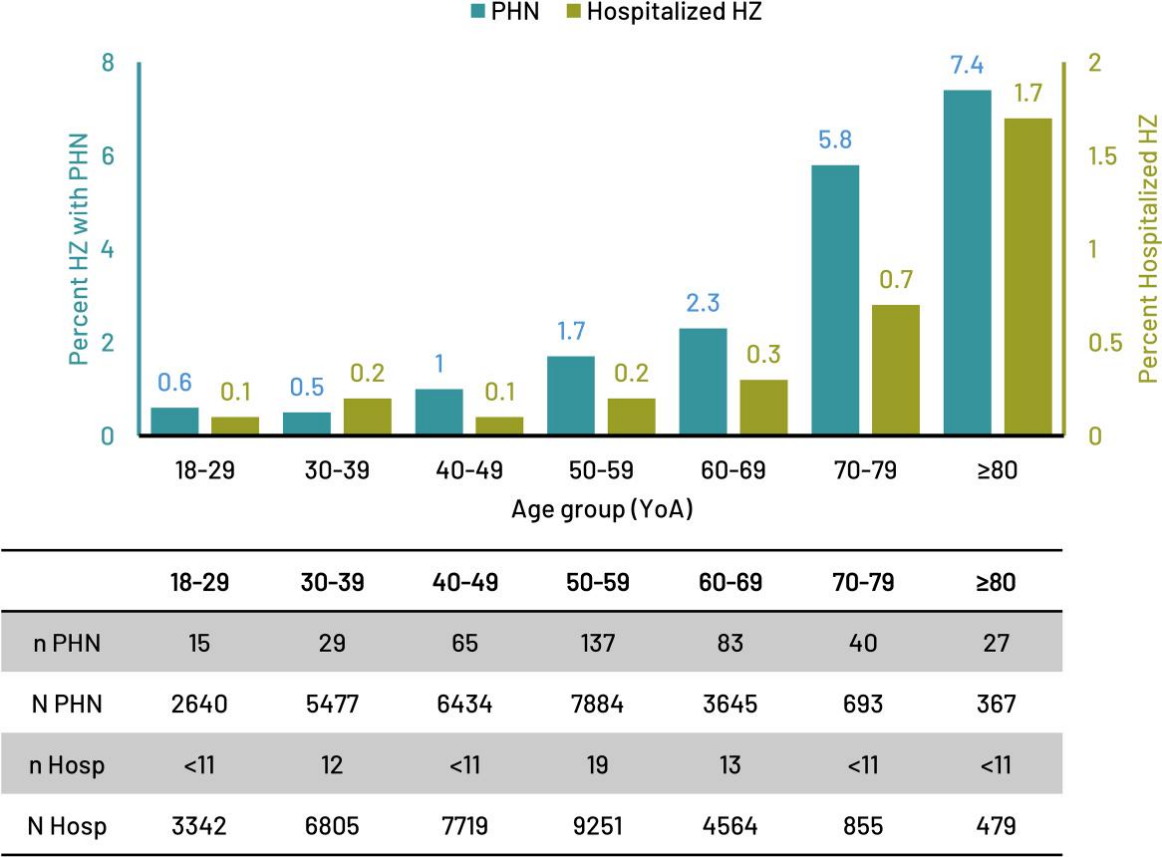
Percent of HZ cases with PHN and/or HZ hospitalization by age group. Note: “N Hosp” represents the total number of people with HZ in each age group. “N PHN” represents the total number of people with HZ in each age group with at least 180 days of continuous enrollment following HZ. Abbreviations: Hosp, hospitalized; HZ, herpes zoster; *n*/*N*, number of cases/total number in category; PHN, postherpetic neuralgia; YoA, years-of-age.

The proportions who developed PHN generally appeared higher in older age groups and tended to increase from the age of 40–49 YoA onwards for example, from 1.0% to 7.4% among immunocompetent 40–49 to ≥80 YoA (Figure 3).

The proportions of adults with HZ hospitalizations also generally appeared higher in older age groups and tended to increase from 60 to 69 YoA onwards. Among ≥60 YoA, HZ hospitalization proportions ranged from 0.3% to 1.7% in immunocompetent adults (Figure 3).

### Sensitivity Analyses

**Figure 4.**
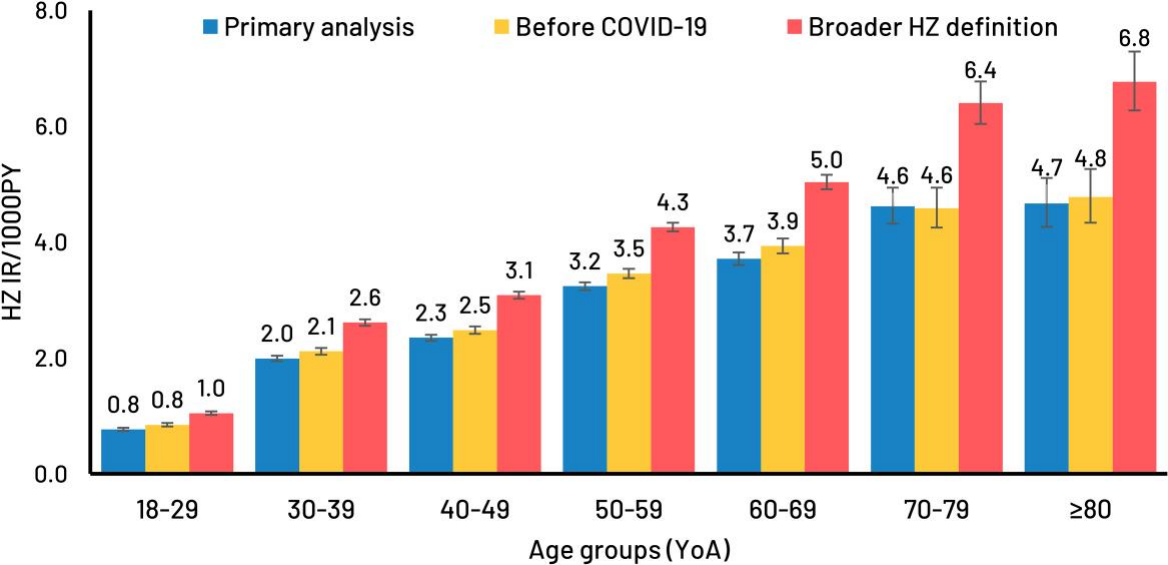
Sensitivity analyses: HZ IR (per 1000PY) in the primary analysis, before COVID-19, and using a broader HZ definition, by age group. Abbreviations: COVID-19, coronavirus disease 2019; HZ, herpes zoster; IR, incidence rate; PY, person-years; YoA, years-of-age.

In a sensitivity analysis using only ICD-10 codes to define HZ cases in the immunocompetent population (instead of ICD-10 codes plus antiviral medication prescriptions within 7 days of HZ diagnosis), the HZ IRs were ∼30% higher, on average, compared across the same age groups (Figure 4). The proportions of HZ cases with PHN and with HZ hospitalization were similar to the main analysis results by age group (see Supplementary Table 1).

The main analysis results were found to be robust in the sensitivity analysis assessing HZ risk in the period before the COVID-19 pandemic, also showing an increase with age and similar HZ risks by age group (Figure 4), with similar proportions of HZ cases with PHN or HZ hospitalization (see Supplementary Table 1).

## DISCUSSION

This observational cohort study from 2015 to 2022 provides estimates of HZ incidence in the US adult (≥18 YoA) immunocompetent population by approximate 10-year age groups, and a comparison of rates to the IR in 50–59 YoA immunocompetent adults. HZ IRs in the age groups 30–39 and 40–49 YoA were found to be noninferior (using the predetermined margin) to the IR in the 50–59 age group, the youngest immunocompetent age group without comorbid conditions covered under the current age-based RZV vaccination recommendation in the US [9]. These results highlight a large, previously unrecognized burden of HZ cases in younger, immunocompetent populations with no comorbid conditions ie, without clear risk factors for HZ (eg, there were 6805 and 7719 HZ cases among 30–39 and 40–49 YoA, respectively, versus 9251 among 50–59 YoA). HZ risk is known to increase with older age, but the burden of HZ in younger adults is less well recognized. However, given the large size of the population aged 30–49 YoA, there was a considerable absolute number of cases observed in the US immunocompetent population in these ages. The study used stringent definitions for HZ cases and immunocompetent status, therefore, the true incidence of HZ may be higher in the general US immunocompetent population.

As expected, the study findings confirm the risk of HZ increases among immunocompetent individuals with each successive age group, from an IR of 0.77 (18–29 YoA) to 4.67 (≥80 YoA) per 1000PY. Although the younger age groups generally appeared to have lower proportions of HZ cases with PHN or HZ hospitalization, the proportion of hospitalized HZ cases appeared to be similar in 30–39 and 50–59 YoA. In sensitivity analyses using a broader HZ case definition based on ICD-10 codes alone, the HZ IRs were found to be approximately 30% higher, with a corresponding absolute increase in number of hospitalizations and PHN cases.

The HZ IRs estimated in our study were lower than those reported for the same age groups in the immunocompetent population ≥50 YoA in 2011–2015 by Tseng et al. [3]. This could be partly explained by our stricter population definition, as the immunocompetent definition in our study excluded those with comorbid conditions associated with higher HZ incidence, which is stricter than the typical immunocompetent definition. Our study also excluded individuals with a wider range of autoimmune conditions and those on immunosuppressive treatments, and used a stricter definition of HZ than Tseng et al. [3]. The HZ IRs estimated in our study were also lower than those reported for the same age groups in the immunocompetent population in 2011 by Johnson et al. [27]. Although Johnson et al. used a somewhat stringent definition of the immunocompetent population, they also did not exclude those with comorbid conditions; the HZ case definition was also broader than in our study. As a result, the HZ IR findings by Johnson et al. [27] were comparable to those by Tseng et al. [3], for example, HZ IR of 6.21 versus 6.74 in 50–54/50–59 YoA; and HZ IR of 11.61 versus 12.78 in ≥80 YoA. By contrast, HZ IRs in our immunocompetent population without comorbid conditions were 3.24 (50–59 YoA) and 4.67 (≥80 YoA), or, using the broader HZ definition, 4.26 (50–59 YoA) and 6.76 (≥80 YoA). Despite the conservative definitions used in our study, the findings showed an important burden of HZ cases in immunocompetent populations 30–49 YoA without any underlying comorbidities or AIDs and who were not on immunosuppressive medication.

The study provides new and up-to-date evidence that could help inform discussions about potential vaccination needs in younger age groups, and addresses existing gaps in the literature on the risk of HZ in immunocompetent people (without comorbid, immunocompromising, or autoimmune conditions) aged 18–49 YoA. The HZ risk in this population aged 30–49 YoA was statistically noninferior to the risk in the 50–59 YoA immunocompetent population—the population with the lowest risk of HZ that is covered under the current US indication for RZV vaccination [9]. Further research is needed to understand how this HZ case burden translates into clinical and economic burden for these younger, healthy age groups. Despite vaccine recommendations, however, vaccine uptake remains low across all age groups [28], thus HZ continues to place a substantial health burden on adults in the US.

Key strengths of our study include the large study population, the stringent definition of the immunocompetent population that removes comorbid conditions associated with higher HZ incidence, and the findings are generalizable to the US immunocompetent adult population with Commercial/Medicare Supplemental health insurance. Although HZ epidemiology is similar in the US and Europe [6] across these age ranges, the generalizability of this study to other non-US settings may be limited. As the proportion in the US South in our study population is high compared to that from the South in the overall US population, the generalizability of the study by US geographic region may be limited. In addition, the strict definition of immunocompetence used may mean that the results do not align with other common definitions for an immunocompetent population. Some limitations relate to the use of database/claims data, including a lack of generalizability to populations not commercially/Medicare-insured, the necessity of imputing clinical outcomes from data not prepared for research purposes, and possible underreporting of certain events and diagnoses, with no direct data on deaths. It is possible that events or diagnoses that occurred before the baseline period or the study timeframe may be missing from the analysis. There was also possible unmeasured confounding, such as disease severity and disease duration, which might have been associated with subsequent risk of HZ. However, these factors are challenging to measure in administrative claims data or might not have been measured. This study assessed proxies for disease activity (such as medication use and healthcare use for disease severity) to measure confounding.

## CONCLUSIONS

Our study identified substantial HZ incidence in the younger immunocompetent population 30–49 YoA, with IRs that were statistically comparable with the 50–59 YoA immunocompetent population. As expected, the study confirms that HZ burden remains important in adults ≥50 YoA, the population for which RZV is currently licensed and recommended. The new evidence of comparable HZ incidence in immunocompetent 30–49 and 50–59 YoA adults increases our knowledge of the HZ case burden in healthy US adults and can help to inform vaccine policymakers.

## Supplementary Material

ofag153_Supplementary_Data

## Supplementary Data

Supplementary materials are available at *Open Forum Infectious Diseases* online. Consisting of data provided by the authors to benefit the reader, the posted materials are not copyedited and are the sole responsibility of the authors, so questions or comments should be addressed to the corresponding author.

## References

[1] Centers for Disease Control and Prevention (CDC). Clinical overview of shingles (herpes zoster). Available at: https://www.cdc.gov/shingles/hcp/clinical-overview/index.html. Accessed 01-08-2025.

[2] Fashner J, Bell AL. Herpes zoster and postherpetic neuralgia: prevention and management. Am Fam Physician 2011; 83:1432–7. 21671543

[3] Tseng HF, Bruxvoort K, Ackerson B, et al The epidemiology of herpes zoster in immunocompetent, unvaccinated adults ≥50 years old: incidence, complications, hospitalization, mortality, and recurrence. J Infect Dis 2020; 222:798–806. 31830250 10.1093/infdis/jiz652 PMC7399704

[4] Johnson RW, Alvarez-Pasquin MJ, Bijl M, et al Herpes zoster epidemiology, management, and disease and economic burden in Europe: a multidisciplinary perspective. Ther Adv Vaccines 2015; 3:109–20. 26478818 10.1177/2051013615599151 PMC4591524

[5] Schmid DS, Miao C, Leung J, Johnson M, Weinberg A, Levin MJ. Comparative antibody responses to the live-attenuated and recombinant herpes zoster vaccines. J Virol 2021; 95:e00240–21. 33762414 10.1128/JVI.00240-21 PMC8316136

[6] Kawai K, Gebremeskel BG, Acosta CJ. Systematic review of incidence and complications of herpes zoster: towards a global perspective. BMJ Open 2014; 4:e004833. 10.1136/bmjopen-2014-004833 PMC4067812 24916088

[7] Hales CM, Harpaz R, Joesoef MR, Bialek SR. Examination of links between herpes zoster incidence and childhood varicella vaccination. Ann Intern Med 2013; 159:739–45. 24297190 10.7326/0003-4819-159-11-201312030-00006 PMC5719886

[8] Centers for Disease Control and Prevention (CDC) . Update on herpes zoster vaccine: licensure for persons aged 50 through 59 years. MMWR Morb Mortal Wkly Rep 2011; 60:1528. 22071592

[9] Anderson TC, Masters NB, Guo A. Use of recombinant zoster vaccine in immunocompromised adults aged ≥19 years: recommendations of the Advisory Committee on Immunization Practices—United States, 2022. MMWR Morb Mortal Wkly Rep 2022; 71:80–4. 35051134 10.15585/mmwr.mm7103a2 PMC8774159

[10] Chen SY, Suaya JA, Li Q, et al Incidence of herpes zoster in patients with altered immune function. Infection 2014; 42:325–34. 24214127 10.1007/s15010-013-0550-8 PMC3968442

[11] Marra F, Parhar K, Huang B, Vadlamudi N. Risk factors for herpes zoster infection: a meta-analysis. Open Forum Infect Dis 2020; 7:ofaa005. 32010734 10.1093/ofid/ofaa005 PMC6984676

[12] Merative.com. Available at: https://www.merative.com/real-world-evidence/real-world-data-analytics. Accessed 24-01-2025.

[13] Food and Drug Administration (FDA) - Center for Biologics Evaluation and Research (CBER). A structured review of electronic coding algorithms for identifying immunocompromised cohorts using administrative claims and electronic health records - Final report. Available at: https://bestinitiative.org/wp-content/uploads/2022/05/Immunocompromised_Algorithm_Final_Report_2021.pdf. Accessed 27-01-2025.

[14] Hanly JG, Thompson K, Skedgel C. Identification of patients with systemic lupus erythematosus in administrative healthcare databases. Lupus 2014; 23:1377–82. 25057038 10.1177/0961203314543917

[15] Culpepper WJ, Marrie RA, Langer-Gould A, et al Validation of an algorithm for identifying MS cases in administrative health claims datasets. Neurology 2019; 92:e1016–28. 30770432 10.1212/WNL.0000000000007043 PMC6442008

[16] Callhoff J, Albrecht K, Marschall U, Strangfeld A, Hoffmann F. Identification of rheumatoid arthritis in German claims data using different algorithms: validation by cross-sectional patient-reported survey data. Pharmacoepidemiol Drug Saf 2023; 32:517–25. 36349482 10.1002/pds.5562

[17] Weng X, Liu L, Barcellos LF, Allison JE, Herrinton LJ. Clustering of inflammatory bowel disease with immune mediated diseases among members of a northern California-managed care organization. Am J Gastroenterol 2007; 102:1429–35. 17437504 10.1111/j.1572-0241.2007.01215.x

[18] Asgari MM, Wu JJ, Gelfand JM, et al Validity of diagnostic codes and prevalence of psoriasis and psoriatic arthritis in a managed care population, 1996–2009. Pharmacoepidemiol Drug Saf 2013; 22:842–9. 23637091 10.1002/pds.3447 PMC3720770

[19] Lipscombe LL, Hwee J, Webster L, Shah BR, Booth GL, Tu K. Identifying diabetes cases from administrative data: a population-based validation study. BMC Health Serv Res 2018; 18:316. 29720153 10.1186/s12913-018-3148-0 PMC5932874

[20] Smidth M, Sokolowski I, Kærsvang L, Vedsted P. Developing an algorithm to identify people with chronic obstructive pulmonary disease (COPD) using administrative data. BMC Med Inform Decis Mak 2012; 12:38. 22616576 10.1186/1472-6947-12-38 PMC3444358

[21] Solberg LI, Engebretson KI, Sperl-Hillen JM, Hroscikoski MC, Connor O, J P. Are claims data accurate enough to identify patients for performance measures or quality improvement? The case of diabetes, heart disease, and depression. Am J Med Qual 2006; 21:238–45. 16849780 10.1177/1062860606288243

[22] Nissen F, Morales DR, Mullerova H, Smeeth L, Douglas IJ, Quint JK. Validation of asthma recording in the clinical practice research datalink (CPRD). BMJ Open 2017; 7:e017474. 10.1136/bmjopen-2017-017474 PMC5724126 28801439

[23] Ronksley PE, Tonelli M, Quan H, et al Validating a case definition for chronic kidney disease using administrative data. Nephrol Dial Transplant 2012; 27:1826–31. 22015442 10.1093/ndt/gfr598

[24] Zhang JX, Joesoef RM, Bialek S, Wang C, Harpaz R. Association of physical trauma with risk of herpes zoster among medicare beneficiaries in the United States. J Infect Dis 2013; 207:1007–11. 23307932 10.1093/infdis/jis937

[25] Yun H, Yang S, Chen L, et al Risk of herpes zoster in autoimmune and inflammatory diseases: implications for vaccination. Arthritis Rheumatol 2016; 68:2328–37. 26990731 10.1002/art.39670 PMC5396838

[26] Bhavsar A, Lonnet G, Wang C, et al Increased risk of herpes zoster in adults ≥50 years old diagnosed with COVID-19 in the United States. Open Forum Infect Dis 2022; 9:ofac118. 35392454 10.1093/ofid/ofac118 PMC8982770

[27] Johnson BH, Palmer L, Gatwood J, Lenhart G, Kawai K, Acosta CJ. Annual incidence rates of herpes zoster among an immunocompetent population in the United States. BMC Infect Dis 2015; 15:502. 26546419 10.1186/s12879-015-1262-8 PMC4636742

[28] Marcum ZA, Jain P, Embry A, Arakaki B, Estevez I, Viscidi E. Incidence of herpes zoster and postherpetic neuralgia and herpes zoster vaccination uptake in a US administrative claims database. Open Forum Infect Dis 2024; 11:ofae211. 38737423 10.1093/ofid/ofae211 PMC11083623

