# Supplementary material for: Age-specific Risk of Herpes Zoster in Immunocompetent Adults ≥18 Years-of-age—A Retrospective Cohort Study in the United States
Source: Open Forum Infect Dis. 2026 Mar 28;13(4):ofag153. doi: 10.1093/ofid/ofag153 (PMC13059686; doi:10.1093/ofid/ofag153)
Supplement: ofag153_Supplementary_Data [file ofag153_supplementary_data.docx]

# Supplemental material

**Figure S1.** Derivation of the immunocompetent population**.**


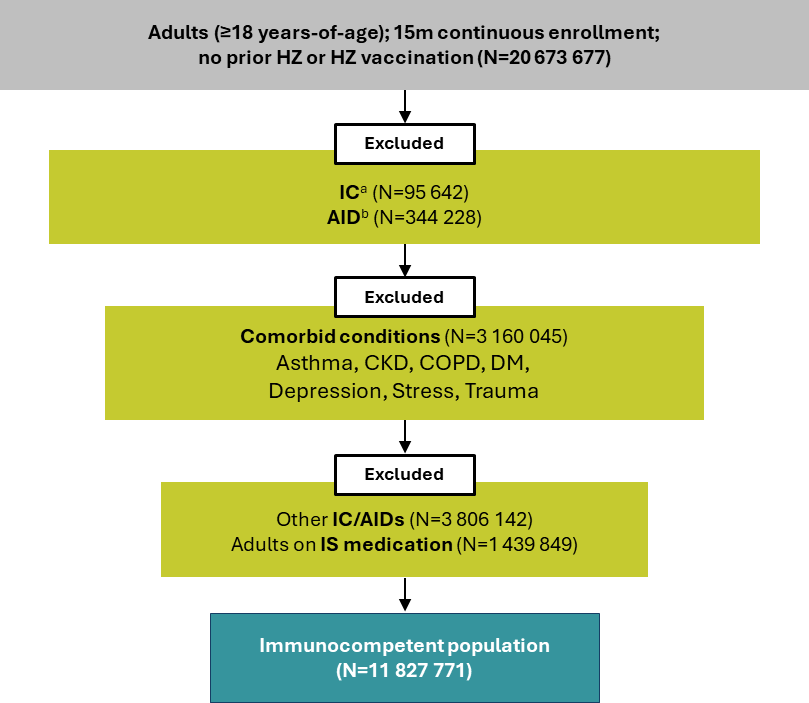


Abbreviations: AID, autoimmune disease; CKD, chronic kidney disease; COPD, chronic obstructive pulmonary disease; DM, diabetes mellitus; HZ, herpes zoster; IC, immunocompromising condition; IS, immunosuppressive; m, months; N, number

^a^ICs: solid tumors, hematologic malignancies, solid organ transplant, human immunodeficiency virus, stem cell transplant; **^b^**AIDs: multiple sclerosis, systemic lupus erythematosus, rheumatoid arthritis, inflammatory bowel disease, psoriasis, psoriatic arthritis

**Alt text:** Graph showing exclusion of 1) common IC and AID populations, 2) common comorbid populations, and 3) additional IC/AID and IS medication populations, in order to obtain the immunocompetent population for analysis from the overall starting population.

**Table S1. Sensitivity analyses: HZ IR per 1000PY (95% CI) and percent of HZ cases with PHN and HZ hospitalization in the primary analysis, before the COVID-19 pandemic, and using a broader HZ definition**

|  | **HZ IR (95% CI) data** | | | | | | | | | | | | |
| --- | --- | --- | --- | --- | --- | --- | --- | --- | --- | --- | --- | --- | --- |
| **Primary analysis** | | | | | | | | | | | | | |
|  | **18–29 YoA** | | **30–39 YoA** | | **40–49 YoA** | | **50–59 YoA** | | **60–69 YoA** | | **70–79 YoA** | | **≥80 YoA** |
| n | 3342 | | 6805 | | 7719 | | 9251 | | 4564 | | 855 | | 479 |
| N | 3 629 744 | | 2 902 845 | | 2 593 703 | | 2 259 519 | | 1 082 484 | | 174 433 | | 99 648 |
| PY | 4 369 363 | | 3 418 681 | | 3 288 415 | | 2 858 838 | | 1 229 766 | | 185 165 | | 102 630 |
| IR | 0.77 | | 1.99 | | 2.35 | | 3.24 | | 3.71 | | 4.62 | | 4.67 |
| 95% CI | 0.74–0.79 | | 1.94–2.04 | | 2.30–2.40 | | 3.17–3.30 | | 3.60–3.82 | | 4.31–4.94 | | 4.26–5.11 |
| **Before COVID-19 pandemic sensitivity analysis** | | | | | | | | | | | | | |
|  | **18–29 YoA** | | **30–39 YoA** | | **40–49 YoA** | | **50–59 YoA** | | **60–69 YoA** | | **70–79 YoA** | | **≥80 YoA** |
| n | 2767 | | 5120 | | 5997 | | 7482 | | 3692 | | 692 | | 420 |
| N | 2 967 580 | | 2 312 756 | | 2 152 932 | | 1 925 744 | | 931 142 | | 154 906 | | 91 153 |
| PY | 3 267 507 | | 2 422 348 | | 2 418 947 | | 2 165 194 | | 939 384 | | 150 927 | | 87 881 |
| IR | 0.85 | | 2.11 | | 2.48 | | 3.46 | | 3.93 | | 4.59 | | 4.78 |
| 95% CI | 0.82–0.88 | | 2.06–2.17 | | 2.42–2.54 | | 3.38–3.54 | | 3.80–4.06 | | 4.25–4.94 | | 4.33–5.26 |
| **Broader HZ sensitivity analysis** | | | | | | | | | | | | | |
|  | **18–29 YoA** | | **30–39 YoA** | | **40–49 YoA** | | **50–59 YoA** | | **60–69 YoA** | | **70–79 YoA** | | **≥80 YoA** |
| n | 4574 | | 8919 | | 10 120 | | 12 154 | | 6181 | | 1183 | | 693 |
| N | 3 629 744 | | 2 902 719 | | 2 593 449 | | 2 259 232 | | 1 082 194 | | 174 415 | | 99 632 |
| PY | 4 368 233 | | 3 416 477 | | 3 285 696 | | 2 855 593 | | 1 227 960 | | 184 918 | | 102 454 |
| IR | 1.05 | | 2.61 | | 3.08 | | 4.26 | | 5.03 | | 6.40 | | 6.76 |
| 95% CI | 1.02–1.08 | | 2.56–2.67 | | 3.02–3.14 | | 4.18–4.33 | | 4.91–5.16 | | 6.04–6.77 | | 6.27–7.29 |
|  | | **% with PHN** | | | | | | **% Hospitalized HZ** | | | | | |
|  | | **Primary analysis** | | **Before COVID-19** | | **Broader HZ definition** | | **Primary analysis** | | **Before COVID-19** | | **Broader HZ definition** | |
| **18–29** | | 0.6 | | 0.5 | | 0.5 | | 0.1 | | 0.1 | | 0.3 | |
| **30–39** | | 0.5 | | 0.6 | | 0.6 | | 0.2 | | 0.2 | | 0.2 | |
| **40–49** | | 1.0 | | 0.9 | | 1.0 | | 0.1 | | 0.1 | | 0.2 | |
| **50–59** | | 1.7 | | 1.5 | | 1.6 | | 0.2 | | 0.2 | | 0.3 | |
| **60–69** | | 2.3 | | 2.5 | | 2.2 | | 0.3 | | 0.3 | | 0.5 | |
| **70–79** | | 5.8 | | 5.3 | | 5.4 | | 0.7 | | 0.7 | | 0.9 | |
| **≥80** | | 7.4 | | 6.8 | | 7.5 | | 1.7 | | 1.7 | | 4.5 | |

Abbreviations: 95% CI, 95% confidence interval; HZ, herpes zoster; IR, incidence rate; n/N, number; PHN, postherpetic neuralgia; PY, person-years; YoA, years-of-age
